# Systemic inflammation down-regulates glyoxalase-1 expression: an experimental study in healthy males

**DOI:** 10.1042/BSR20210954

**Published:** 2021-07-02

**Authors:** Rob G.H. Driessen, Dorien Kiers, Casper G. Schalkwijk, Jean L.J.M. Scheijen, Jelle Gerretsen, Peter Pickkers, Marcel C.G. van de Poll, Iwan C.C. van der Horst, Dennis C.J.J. Bergmans, Matthijs Kox, Bas C.T. van Bussel

**Affiliations:** 1 Department of Intensive Care Medicine, Maastricht University Medical Center +, Maastricht, The Netherlands; 2 Department of Cardiology, Maastricht University Medical Center +, Maastricht, The Netherlands; 3 Department of Intensive Care Medicine, Radboud University Medical Center, Nijmegen, The Netherlands; 4 Department of Internal Medicine, Maastricht University Medical Center +, Maastricht, The Netherlands; 5 Cardiovascular Research Institute Maastricht (CARIM), Maastricht, The Netherlands; 6 School of Nutrition and Translational Research in Metabolism (NUTRIM), Maastricht, The Netherlands; 7 Department of Surgery, Maastricht University Medical Center +, Maastricht, The Netherlands; 8 Care and Public Health Research Institute (CAPHRI), Maastricht, The Netherlands

**Keywords:** dicarbonyl, glyoxalase, hypoxia, inflammation, methylglyoxal, sepsis

## Abstract

Background: Hypoxia and inflammation are hallmarks of critical illness, related to multiple organ failure. A possible mechanism leading to multiple organ failure is hypoxia- or inflammation-induced down-regulation of the detoxifying glyoxalase system that clears dicarbonyl stress. The dicarbonyl methylglyoxal (MGO) is a highly reactive agent produced by metabolic pathways such as anaerobic glycolysis and gluconeogenesis. MGO leads to protein damage and ultimately multi-organ failure. Whether detoxification of MGO into D-lactate by glyoxalase functions appropriately under conditions of hypoxia and inflammation is largely unknown. We investigated the effect of inflammation and hypoxia on the MGO pathway in humans *in vivo.*

Methods: After prehydration with glucose 2.5% solution, ten healthy males were exposed to hypoxia (arterial saturation 80–85%) for 3.5 h using an air-tight respiratory helmet, ten males to experimental endotoxemia (LPS 2 ng/kg i.v.), ten males to LPS+hypoxia and ten males to none of these interventions (control group). Serial blood samples were drawn, and glyoxalase-1 mRNA expression, MGO, methylglyoxal-derived hydroimidazolone-1 (MG-H1), D-lactate and L-lactate levels, were measured serially.

Results: Glyoxalase-1 mRNA expression decreased in the LPS (β (95%CI); -0.87 (-1.24; -0.50) and the LPS+hypoxia groups; -0.78 (-1.07; -0.48) (*P*<0.001). MGO was equal between groups, whereas MG-H1 increased over time in the control group only (*P*=0.003). D-Lactate was increased in all four groups. L-Lactate was increased in all groups, except in the control group.

Conclusion: Systemic inflammation downregulates glyoxalase-1 mRNA expression in humans. This is a possible mechanism leading to cell damage and multi-organ failure in critical illness with potential for intervention.

## Introduction

Severe inflammatory conditions, such as sepsis, leading to multiple organ failure (MOF), are still a major challenge in intensive care units (ICUs) [1,2]. Hypoxia is another hallmark of critical illness and sepsis, interacting with severe inflammation at a cellular level causing cytopathic hypoxia [3]. Several pathobiological mechanisms involved in the development of MOF, such as inflammation [4], coagulation [5], endothelial dysfunction [6] and oxidative stress [7], have been investigated previously. However, little attention has been paid to another possibly relevant mechanism, the detoxifying glyoxalase system that clears dicarbonyl stress.

It has been postulated that increased formation of the dicarbonyls methylglyoxal (MGO), glyoxal (GO) and 3-deoxyglucosone (3-DG), induced by inflammation and hypoxia, may contribute to multi-organ failure in critical illness [8]. These reactive dicarbonyls are produced by several metabolic pathways, such as anaerobic glycolysis and gluconeogenesis [9]. Inflammation leads to a switch from oxidative phosphorylation to glycolysis, which may drive production of dicarbonyls [10]. Hypoxia leads to cellular adaptation to low oxygen by activation of hypoxia-inducible factors (HIFs) and activation of anaerobic glycolysis, which also may drive dicarbonyl production [11]. The produced dicarbonyls damage intracellular and extracellular proteins mainly due to arginine modifications and the formation of methylglyoxal derived hydroimidazolone-1 (MG-H1), leading to cell and tissue dysfunction [12], which has been shown to impair organ function [13–16].

The glyoxalase system clears dicarbonyl stress by detoxifying methylglyoxal, presumably the most reactive and damaging dicarbonyl [17]. It does so by converting methylglyoxal (MGO) into D-lactate, with glyoxalase-1 (GLO-1) as the key enzyme involved [18]. D-Lactate concentrations thereby serve as a reflection of cumulative MGO exposure. The glyoxalase system is of particular interest because it has potential for therapeutic intervention by either lowering MGO by arginine or pyridoxamine [18] or by up-regulating the glyoxalase-system with isothiocyanate[19]. However, whether detoxification of MGO by GLO-1 functions appropriately during systemic inflammation and/or hypoxia in humans is largely unknown [20]. We hypothesize that inflammation and hypoxia increase MGO, D-lactate and MG-H1 in humans through reduced GLO-1 expression.

Herein, we investigated the effects of systemic inflammation induced by experimental endotoxemia and hypoxia on GLO-1 expression, MGO, D-lactate and MG-H1 in healthy males.

## Methods

### Participants

Data of a total of 40 healthy, non-smoking males, aged 18-29 years are described in the present study, who took part in three randomized studies registered at Clinicaltrials.gov (NCT01889823, NCT01978158, and NCT02642237). Data from the hypoxia group (*n*=10) were obtained from the study registered under NCT01889823 [21], data from the LPS (*n*=10) and LPS+hypoxia (*n*=10) groups from the study registered under NCT01978158 [21], and data from the control group (*n*=10) from the study registered under NCT02642237 [22]. All studies were approved by the local medical ethics committee (CMO Arnhem-Nijmegen), and written informed consent was obtained from all participants. All study procedures were in accordance with the declaration of Helsinki. Participants were screened before the start of the study and had a normal physical examination, electrocardiography and routine laboratory values. Participants with a pre-existent disease or febrile illness within 4 weeks before the experiment were excluded. Participants were asked to refrain from caffeine and alcohol intake in the preceding 24 h and food in the preceding 12 h, before the experiment. Height and weight were measured and recorded.

### Study design

**Figure 1:**
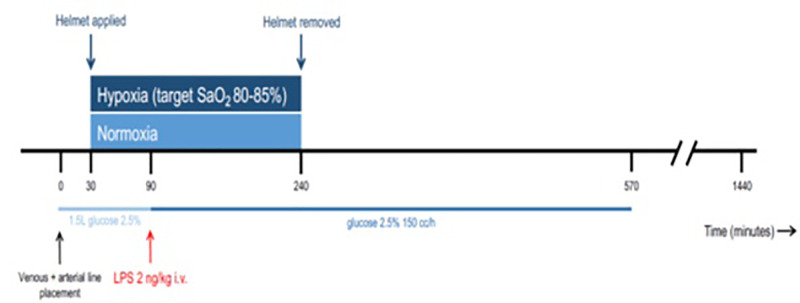
Overview of the human endotoxemia and hypoxia model procedures First, venous and arterial cannula were placed. Subsequently, prehydration with 1.5 L glucose 2.5% infusion (light blue line) was started. After 1 h, prehydration was ceased, and maintenance fluid infusion of the same solution of 150 ml/h was commenced. Application of a non-invasive helmet for 210 min (blue arrows) to induce hypoxia or normoxia (indicated by the blue square) was initiated after 30 min. One hour after the start of prehydration and application of the helmet, 2 ng/kg LPS was administered intravenously (red arrow). Blood samples were drawn at ten time points from 0 until 570 min, and an additional blood sample was drawn after 24 h (1440 min).

The study design with timing and duration of interventions is shown in Figure 1. Study procedures were identical in all four groups except the intervention and slightly different time points for blood withdrawal in the control group. Ten participants were exposed to hypoxia for 3.5 h by titration of FiO_2_ to a peripheral saturation (SaO_2_) of 80–85% using a nitrogen/medical air mixture and an air-tight respiratory helmet (CaStar, Starmed, Italy) (hypoxia group). A systemic inflammatory response was elicited in ten participants by the administration of 2 ng/kg U.S. Reference Escherichia coli endotoxin (serotype O:113, Clinical Center Reference endotoxin, National Institute of Health, Bethesda, U.S.A.) (LPS group). This human endotoxemia model has been successfully applied as a translational model for sepsis and gives a short-lived, controlled inflammatory response clinically causing fever, tachycardia and mild hypotension as well as leucocytosis and increased plasma cytokine levels [23–26]. Ten participants were exposed to hypoxia and LPS, with the LPS administered one hour after hypoxia initiation (LPS+hypoxia group). Finally, ten participants underwent the same study protocol as described below; however, these participants were not exposed to LPS or hypoxia (control group). Although the original study by Koch et al. investigated the effect of endotoxin tolerance with a live-attenuated Influenza vaccine, the blood samples used in the present study were taken only from participants receiving placebo (no LPS) [22].

### Procedures and recording of vital signs

A venous cannula was placed for fluid infusion. Patients received prehydration with 1.5 L 2.5% glucose/0.45% saline in the hour preceding endotoxin administration, followed by hydration with 150 ml/h of the same solution for 6 h. The experimental endotoxemia protocol required an infusion of fluids due to vasodilation and the risk of hypotension. In total 120 mg of glucose was infused during the study protocol. An arterial cannula in the radial artery facilitated blood pressure monitoring and blood withdrawal. Blood was drawn at 10 different points in time; for the hypoxia, LPS and LPS+hypoxia groups: 0, 90, 150, 180, 210, 240, 270, 330, 450, 570 and 1440 min (24 h); for the control group: 90, 150, 180, 210, 270 and 330, 450 and 570 min. Unfortunately, no baseline samples of the control group participants were available. Blood samples were collected in Paxgene blood RNA tubes (Qiagen®) and lithium heparin blood tubes. Plasma was separated by centrifugation at 2000 ***g*** for 10 min at 4°C. Samples were stored at -80°C until analysis. Heart rate was monitored using a three-lead electrocardiogram, and SaO_2_ was monitored using a pulse oximeter. Body temperature was measured using an infrared tympanic thermometer (FirstTemp Genius 2; Covidien, Ireland) every 30 min. Leukocyte counts were measured using routine analysis methods also used for patient samples (flow cytometric analysis on a Sysmex XE-5000). Plasma cytokines were measured by simultaneous Luminex assays (hypoxia, LPS and LPS+hypoxia groups: Milliplex, Merck Millipore; Billerica, USA; control group: R&D systems; Abingdon Science Park, UK).

### Glyoxalase-1 (GLO1) mRNA expression

To determine GLO1 mRNA expression in leukocytes, whole blood was obtained in Paxgene vacutainer tubes (Qiagen, Venlo, the Netherlands). These tubes contain a solution which mixes with blood immediately upon withdrawal, lyses the cells, and stabilizes the RNA, after which tubes were stored at -80°C for subsequent RNA isolation [27]. Samples for this analysis were obtained from the hypoxia, LPS and LPS+hypoxia groups at *t* = 0, 90, 150, 240, 270, 330, 450 and 1440 min. RNA was isolated batch wise using the Paxgene blood RNA kit (Qiagen, Venlo, the Netherlands). The iScript cDNA synthesis kit (Bio-Rad laboratories, Lunteren, the Netherlands) was used to convert RNA into cDNA. Quantitative PCR (qPCR) was performed on aCFX96™ Real-Time System (Bio-Rad laboratories, Lunteren, the Netherlands using the following TaqMan primer-probe pairs; human GLO1 Hs00198702_m1 and the reference (housekeeping) gen human RPL27 Hs03044961_g1 (Life technologies, Darmstadt, Germany). We determined GLO1 mRNA expression in the hypoxia, LPS and the LPS+hypoxia group. There was no mRNA available for the participants in the control group.

### Methylglyoxal (MGO)

Plasma concentration of MGO was measured in plasma samples for the hypoxia, LPS and LPS+hypoxia groups at *t* = 0, 90, 150, 180, 210, 240, 270, 330, 450, 570 and 1440 min and for the control group at *t* = 90, 150, 180, 210, 270, 330, 450 and 570 min. Reversed-phase ultra-performance liquid chromatography-tandem mass spectrometry was used to measure MGO concentrations, as described earlier [28]. The inter-assay variation for MGO was 6.0%.

### D-lactate, L-lactate and Nδ-(5-hydro-5-methyl-4-imidazolone-2-yl)-ornithine (MG-H1)

Plasma D- and L-lactate were measured at *t* = 0 and 450 min in the hypoxia, LPS, LPS+hypoxia, and the control group with ultra-performance liquid chromatography mass spectrometry with labelled internal standard [29]. MG-H1 was measured at these same points in time by ultra-performance liquid chromatography-tandem mass spectrometry [30].

### Statistical analysis

Data analyses were performed with SPSS (Statistical Package for Social Sciences, version 24, IBM Corporation, U.S.A.), STATA (Data Analyses and Statistical Software, version 13, StataCorp LLC, U.S.A.) and GraphPad Prism version 5.00 for Windows (GraphPad Software, U.S.A.). Data are presented as mean ± standard error of the mean (SEM). Means were compared using one-way ANOVA and Student’s *t*-tests for paired samples, as appropriate.

We used generalized estimating equations (GEE, using STATA) to investigate longitudinal regression coefficients (β) with their 95% confidence intervals (95%CI) that represent the difference in the development of GLO1 mRNA expression, and MGO plasma concentration over time between the experimental conditions. An exchangeable correlation structure was used. Crude models were adjusted for age and body mass index. GEE has several advantages over, for example, ANOVA for repeated measures to analyze longitudinal data. GEE reports an effect estimate with 95% CI, allows analyses of unequally spaced time intervals, and handles missing data robustly by analyzing all available data. Results were adjusted for age and body-mass index to minimize any effect of random variation between the participants. A two-sided *P*-value <0.05 was considered statistically significant.

## Results

General characteristics of the 40 males, mean age of 22 ± 2 years, were similar across experimental groups (Table 1).

**Table 1:** Baseline characteristics of 40 healthy male participants

	Experimental conditions			Control	
	Hypoxia (*n*=10)	LPS (*n*=10)	LPS+hypoxia (*n*=10)	(*n*=10)	*P*-value
Age, years	21 ± 2	21 ± 2	21 ± 2	22 ± 2	0.598
Height, cm	183 ± 5	185 ± 8	184 ± 7	184 ± 7	0.976
Weight, kg	78 ± 11	77 ± 10	77 ± 9	77 ± 12	0.999
Body mass index, kg/m^2^	23 ± 3	22 ± 2	23 ± 3	23 ± 3	0.970
Body surface area, m^2^	2 ± 0.2	2 ± 0.2	2 ± 0.1	2 ± 0.2	0.999

Data are means ± standard deviation; *P*-values by one-way ANOVA

LPS, lipopolysaccharide

### Inflammatory parameters

An extensive description of inflammatory parameters in each group is provided elsewhere [22,31]. In Table 2, baseline and peak values (post-LPS for the appropriate groups and at the corresponding timepoints in the non-LPS groups) of leukocyte counts and body temperature as well as peak plasma levels of the pro-inflammatory cytokines TNF-α and IL-6 are listed.

**Table 2:** Inflammatory parameters in the 40 male participants

	Experimental conditions						Control	
	Hypoxia baseline (*n*=10)	Hypoxia peak (*n*=10)	LPS baseline (*n*=10)	LPS peak (*n*=10)	LPS+hypoxia baseline (*n*=10)	LPS+hypoxia peak (*n*=10)	Baseline (*n*=10)	Peak (*n*=10)
Leukocytes, ×10^9^/l	5.5 ± 1.2	7.8 ± 1.4	4.9 ±1.1	11.0 ± 1.9	5.2 ± 1.1	14.5 ± 1.6	6.2 ± 1.6	6.2 ± 1.3
Body temperature, °C	36.4 ± 0.3	36.6 ± 0.4	36.6 ± 0.3	37.9 ± 0.6	36.8 ± 0.3	38.3 ± 0.6	36.7 ± 0.4	37.0 ± 0.4
Plasma TNF-α, pg/mL	5.5 ± 2.9	5.0 ± 2.7	3.8 ± 0.7	284.6 ± 135.1	3.9 ± 0.9	195.5 ± 77.0	4.6 ± 1.9	4.1 ± 1.7
Plasma IL-6, pg/ml	3.2 ± 0.0	3.2 ± 0.0	3.2 ± 0.0	478.6 ± 241.3	3.2 ± 0.0	292.3 ± 156.4	1.9 ± 0.4	1.8 ± 0.1

Data are means ± standard deviation.

LPS, lipopolysaccharide

Peak was measured at 360, 180, 90 and 120 min post-LPS administration for leukocytes, body temperature, TNF-α and IL-6, respectively.

### GLO1 mRNA expression

**Figure 2:**
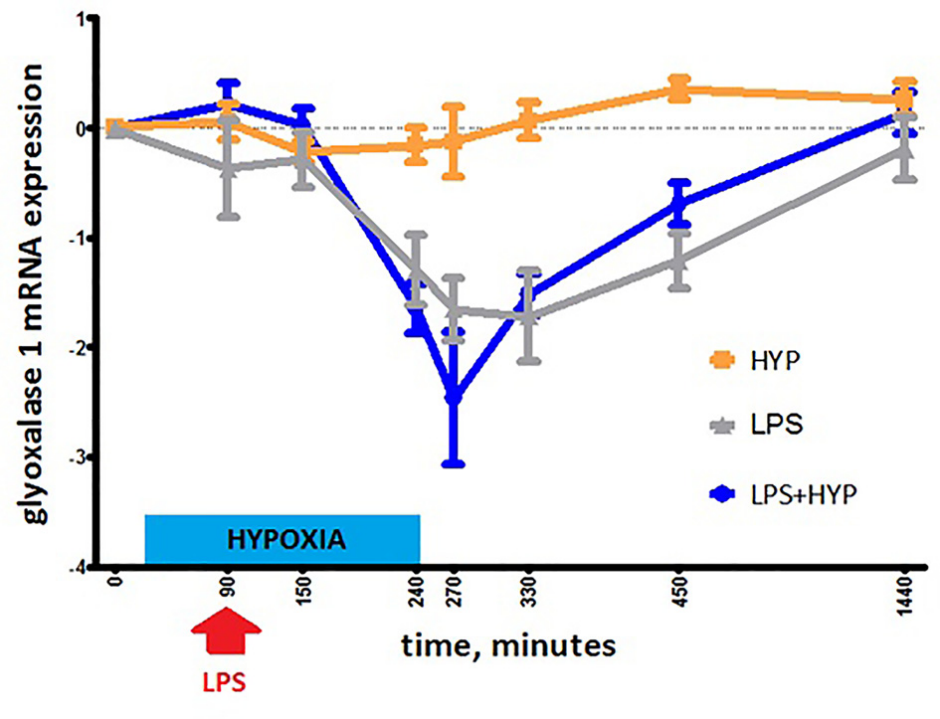
Glyoxalase-1 mRNA expression in the hypoxia (HYP, orange line), LPS (grey line), and LPS+hypoxia (LPS+HYP, blue line) groups during the experiment, depicted as means + standard error of the mean After adjustment for age and BMI, GLO1 expression decreased in the LPS (β (95%CI); -0.87 (-1.24; -0.50)) and LPS+hypoxia (-0.78 (-1.07; -0.48)) groups, compared with the hypoxia group (*P*<0.001), calculated using generalized estimating equations.

After adjustment for age and BMI, GLO1 expression decreased in the LPS (β (95%CI); -0.87 (-1.24; -0.50)) and LPS+hypoxia (-0.78 (-1.07; -0.48)) groups over time, compared with the hypoxia group (*P*<0.001, Figure 2).

### MGO concentration

**Figure 3:**
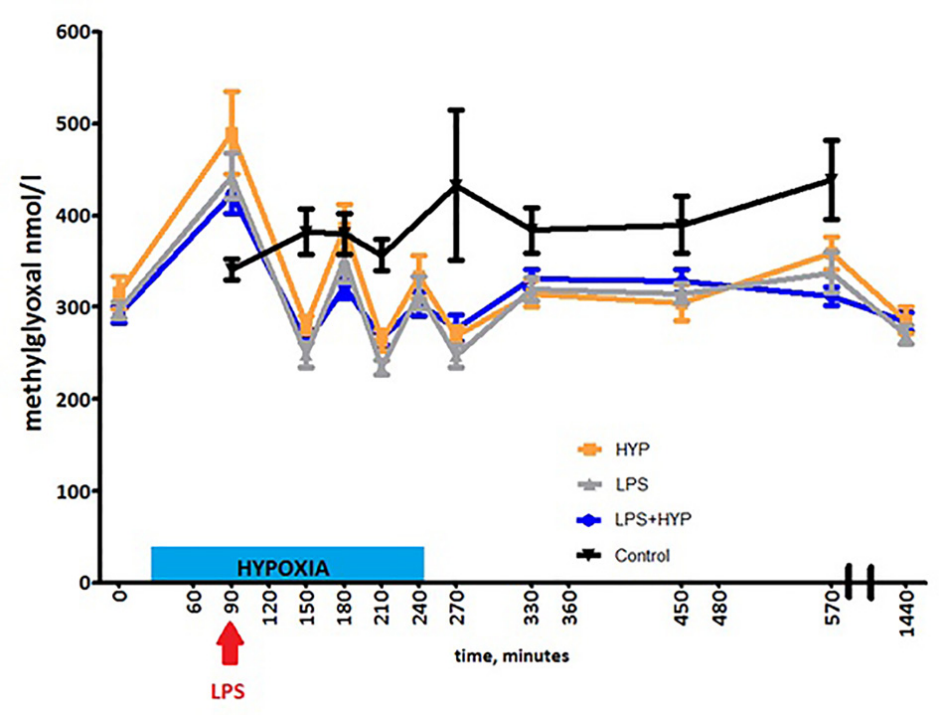
Methylglyoxal (MGO) concentrations for the hypoxia (orange line), LPS (grey line), combined LPS and hypoxia (blue line), and control (black line) groups during the experiment depicted as means + standard error of the mean No between-group differences were found using generalized estimating equations.

After adjustment for age and BMI, MGO concentrations did not differ over time between hypoxia, LPS, and the LPS+hypoxia group (*P*-values 0.902 for LPS and 0.172 for hypoxia versus LPS+hypoxia). MGO levels peaked at *t* = 90 min (*P*<0.001) (Figure 3) in all three groups, before LPS was administered. MGO also tended to increase over time in the control group, albeit not statistically different (*P*=0.066). The control group peak was observed later compared with the other groups (*t* = 270 min, Figure 3).

### D-Lactate, L-lactate, and MG-H1 levels

**Figure 4:**
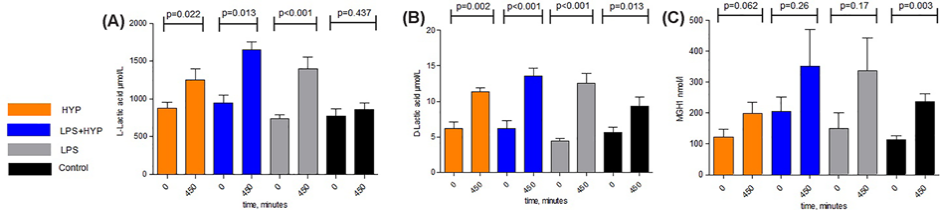
Concentrations of L- and D-lactate and MG-H1 for the different conditions and the control group. A: Panel A: L-lactate, B: Panel B: D-lactate, C: Panel C: MG-H1 L- (panel A) and D-lactate (panel B), and MG-H1 (panel C) concentrations in the hypoxia (orange boxes), LPS (grey boxes), combined LPS and hypoxia (blue boxes), and the control group (black boxes) at 0 and 450 min. Results are reported as means + standard error of the mean (SEM). Student’s *t*-test was used for determining *P*-values.

D-Lactate, the product of MGO broken down by glyoxalase, increased significantly between 0 and 450 min in all the intervention groups (hypoxia: *P*=0.002, LPS: *P*<0.001 and LPS+hypoxia: *P*<0.001), but also in the control group (*P*=0.013) (Figure 4). L-lactate levels, an end-product of glucose metabolism and also a marker for tissue hypoxia, increased over time in the hypoxia (*P*=0.022), LPS (*P*<0.001) and LPS+hypoxia group (*P*=0.013), but not in the control group (*P*=0.437). MG-H1, the major advanced glycation end product (AGE) of MGO, did not significantly change over time within the intervention groups (hypoxia: *P*=0.062, LPS: *P*=0.17 and LPS+hypoxia *P*=0.26). A trend might be observed suggesting an increase in MG-H1 over time in the three experimental groups; however, MG-H1 levels also increased over time in the control group (*P*=0.003) (Figure 4).

## Discussion

This experimental study in healthy males yields two main findings. First, a significant downregulation of GLO-1-expression was identified in response to inflammation, but not hypoxia. Second, experimental hypoxia and inflammation did not lead to a relevant and unequivocal difference in MGO and MG-H1 concentrations over time between the conditions.

We comprehensively investigated the effect of both inflammation and hypoxia on the dicarbonyl stress pathway in humans. Previously, *in vitro* research described a cascade linking inflammation to reduced GLO-1 expression and accumulation of both MGO and AGEs in ruptured human carotid plaques [32]. Furthermore, in mice, it was shown that increased MGO levels augmented vascular inflammation partially independent of hyperglycemia [33]. In a case–control study in sepsis patients, MGO was higher at sepsis onset and after 24 h compared with controls, and MGO was an early predictor for survival in these patients [20]. GLO-1 was reduced in patients with septic shock after 24 h. However, no measurements of D-lactate or MG-H1 were conducted, and the effect of hypoxia on the dicarbonyl pathway was not investigated.

In the present study, GLO-1 expression was significantly downregulated in healthy males receiving LPS but was not influenced by hypoxia. The glyoxalase detoxifying system with GLO-1 as its key enzyme depends on the presence of glutathione [18]. Inflammatory conditions are associated with an increased state of oxidative stress, affecting glutathione and inhibiting the cytosolic glutathione-dependent glyoxalase system [34]. This could lead to an accumulation of dicarbonyls. However, in these healthy men, the GLO-1 expression normalized during the 8-hour experiment, reflecting a transient effect of endotoxemia. The swift recovery of the glyoxalase system in healthy volunteers could explain why MGO blood concentrations were not higher in the LPS groups compared with the control group and suggest intact compensation mechanisms. Moreover, the endotoxemia model does not fully resemble a full-blown septic shock state in which, as alluded to before, increased MGO concentrations were found [20]. Because we used Paxgene tubes for the determination of GLO-1 mRNA expression, which result in immediate lysis of leukocytes and stabilization of RNA following blood withdrawal, leukocyte viability was not an issue, and stability of RNA stored in these tubes was previously shown to be excellent [27]. Furthermore, the LPS dosage given to the participants in this experiment is not expected to cause cell death or apoptosis.

Although MGO concentrations peaked between 0 and 90 min in all conditions in our experiment, there were no differences between the three experimental conditions. This peak in MGO concentration occurred before LPS administration and was also present in the LPS group (with normoxia) and thus can neither be explained by hypoxia nor by inflammation. The effect of the prehydration with 1.5 L 2.5% glucose/0.45% saline (i.e. glucose infusion) could play a role in this peak and the observed increase in D-lactate as a breakdown product of MGO, as this increase over time was also observed in the control experiment. Indeed, previous research has demonstrated that dicarbonyl concentrations increase during an oral glucose tolerance test, even in individuals with normal glucose metabolism [35].

Post-translational modification of proteins, forming advanced glycation end products, is an important consequence of increased dicarbonyl stress. Previous studies have pointed out that MG-H1 is a major MGO protein modification product in humans [36] and is considered a key pathway leading to hyperglycemia-induced complications of diabetes mellitus [12,37]. To our knowledge, no previous studies are investigating MG-H1 concentrations in inflammatory states *in vivo*. Although in the present study, a trend in increase in MG-H1 concentrations was present, this result was not statistically significant for the experimental conditions possibly due to the small sample size.

The study has several strengths and limitations. First, we used a homogenous study population consisting of healthy males. Furthermore, all the study participants underwent the same standardized study protocol and this human endotoxemia model is described in detail earlier [21] and used in several other studies [22,31,38]. This has the advantage of studying hypoxia and inflammation in humans in a highly standardized way. Notably, glyoxalase expression measurements, dicarbonyls, and their modifications end products were performed using the gold standard techniques intended to investigate this pathway comprehensively [28]. The limitation of the present study, including healthy males only, is that this limits generalizability to women and patients with comorbidities. Data and blood samples from volunteers who took part in three separate studies [21,22] were used for the present investigation and we cannot rule out that this influenced the results. We tried to minimize this effect by adjusting for age and BMI in the four groups, also because aging and obesity are both associated with dicarbonyl stress [9]. Because of limited availability of sample that we could assay, we had to prioritize which components of the dicarbonyl pathway we could measure. For instance, we did not measure glutathione, an important catalysing factor in the glyoxalase pathway. However, as glyoxalase-I is the key-limiting enzyme in this pathway, we believe it is justifiable to emphasize on this enzyme. Furthermore, although applied in several earlier studies [22,31,38] the human endotoxemia model does not entirely resemble a full-blown sepsis state observed in critically ill patients. This might have caused an underestimation of the effects of hypoxia and inflammation on dicarbonyl stress and may explain that we did not observe the hypothesized increase in MGO concentration after GLO-1 decrease. Nevertheless, the significant peak in MGO concentration occurring early in the experimental groups suggests that our study was sensitive to reveal any effect of LPS and hypoxia on MGO, if present, in healthy males. Therefore, with regards to our initial hypothesis, the lack of effect on MGO and MG-H1 of hypoxia and inflammation can be regarded as a negative result of this study. Because Paxgene samples of the control experiment were not available, we cannot entirely exclude that we missed a downregulating effect of hypoxia on GLO-1 expression. In fact prehydration with 1.5 L 2.5% glucose/0.45% saline (i.e., glucose infusion) could have biased the effect of hypoxia on GLO-1 expression toward zero as it has been shown that hyperglycemia up-regulates GLO-1-expression [39]. Furthermore, we did not collect blood cells for functional assays, which limits further investigation of GLO-1 activity. In addition, the experiment cannot exclude that D- and L-lactate levels were determined by intercurrent changes in gut permeability caused by LPS or hypoxia [8,40]. Nevertheless, given the paucity of human *in vivo* data on this pathway, our study provides valuable insights into the interactions between inflammation, hypoxia and dicarbonyl stress. This is important as therapeutic options by enhancing glyoxalase activity by a combination of trans-resveratrol and hesperitin showed to reduce methylglyoxal and protein modifications by MGO in overweight and obese individuals [41]. Other therapeutic options are GLO-1 induction by isothiocyanates [19] or scavenging MGO by pyridoxamine or arginine [18] to lower toxic dicarbonyl stress.

In conclusion, our study shows that systemic inflammation downregulates GLO-1 in humans. Down-regulation of GLO-1 is a possible mechanism leading to cell damage and multi-organ failure in sepsis with intervention potential. We did not observe significant differences in MGO concentrations in healthy males. The results urge further investigation of the glyoxalase pathway in sepsis.

## Perspectives

- Hypoxia and inflammation may lead to multiple organ failure in critical illness due to downregulation of the detoxifying enzyme glyoxalase, which clears the highly reactive and protein damaging dicarbonyl methylglyoxal.
- In the present study, glyoxalase-1 mRNA expression was significantly down-regulated by induced inflammation, but not by hypoxia, in humans.
- Down-regulation of GLO-1 is a possible mechanism leading to cell damage and multi-organ failure in sepsis with intervention potential, urging further investigation of the glyoxalase pathway in sepsis.

## Data Availability

All data will be made available on request.

## Abbreviations

3-DG: 3-deoxyglucosone
AGE: advanced glycation end product
GLO1: glyoxalase-1
GO: glyoxal
HIF: hypoxia-inducible factor
LPS: lipopolysaccharide
MGO: methylglyoxal
